# Expanding the neurodevelopmental phenotypes of individuals with de novo *KMT2A* variants

**DOI:** 10.1038/s41525-019-0083-x

**Published:** 2019-04-26

**Authors:** Ada J. S. Chan, Cheryl Cytrynbaum, Ny Hoang, Patricia M. Ambrozewicz, Rosanna Weksberg, Irene Drmic, Anne Ritzema, Russell Schachar, Susan Walker, Mohammed Uddin, Mehdi Zarrei, Ryan K. C. Yuen, Stephen W. Scherer

**Affiliations:** 1 The Centre for Applied Genomics, Genetics and Genome Biology, The Hospital for Sick Children, Toronto, ON Canada; 2 Department of Molecular Genetics, University of Toronto, Toronto, ON Canada; 3 Division of Clinical and Metabolic Genetics, Department of Pediatrics, The Hospital for Sick Children, Toronto, ON Canada; 4 Program in Genetics and Genome Biology, The Hospital for Sick Children, Toronto, ON Canada; 5 Department of Genetic Counselling, The Hospital for Sick Children, Toronto, ON Canada; 6 Autism Research Unit, The Hospital for Sick Children, Toronto, ON Canada; 7 Department of Psychology, The Hospital for Sick Children, Toronto, ON Canada; 8 Institute of Medical Science, University of Toronto, Toronto, ON Canada; 9 Department of Paediatrics, University of Toronto, Toronto, ON Canada; 10 Ron Joyce Children’s Health Centre, Hamilton Health Services, Hamilton, ON Canada; 11 Department of Psychiatry, The Hospital for Sick Children, Toronto, ON Canada; 12 Department of Psychiatry, University of Toronto, Toronto, ON Canada; 13 College of Medicine, Mohammed Bin Rashid University of Medicine and Health Sciences, Dubai, United Arab Emirates; 14 McLaughin Centre, University of Toronto, Toronto, ON Canada

**Keywords:** Autism spectrum disorders, Disease genetics

## Abstract

De novo loss-of-function (LoF) variants in the *KMT2A* gene are associated with Wiedemann−Steiner Syndrome (WSS). Recently, de novo *KMT2A* variants have been identified in sequencing studies of cohorts of individuals with neurodevelopmental disorders (NDDs). However, most of these studies lack the detailed clinical information required to determine whether those individuals have isolated NDDs or WSS (i.e. syndromic NDDs). We performed thorough clinical and neurodevelopmental phenotyping on six individuals with de novo *KMT2A* variants. From these data, we found that all six patients met clinical criteria for WSS and we further define the neurodevelopmental phenotypes associated with *KMT2A* variants and WSS. In particular, we identified a subtype of Autism Spectrum Disorder (ASD) in five individuals, characterized by marked rigid, repetitive and inflexible behaviours, emotional dysregulation, externalizing behaviours, but relative social motivation. To further explore the clinical spectrum associated with *KMT2A* variants, we also conducted a meta-analysis of individuals with *KMT2A* variants reported in the published literature. We found that de novo LoF or missense variants in *KMT2A* were significantly more prevalent than predicted by a previously established statistical model of de novo mutation rate for *KMT2A*. Our genotype−phenotype findings better define the clinical spectrum associated with *KMT2A* variants and suggest that individuals with de novo LoF and missense variants likely have a clinically unrecognized diagnosis of WSS, rather than isolated NDD or ASD alone. This highlights the importance of a clinical genetic and neurodevelopmental assessment for individuals with such variants in *KMT2A*.

## Introduction

The *KMT2A* gene, also known as *MLL*, is on chr11q23 and encodes an H3K4 methyltransferase enzyme that regulates the expression of other genes including neuronal and homeobox-containing developmental genes.^1^ *KMT2A* is essential for embryonic development, hematopoiesis,^2^ and neural development^3^ and dysregulation of *KMT2A* function is associated with various human disorders. Recurrent somatic translocations between *KMT2A* and 70 different fusion partners result in leukaemia,^4^ whereas germline de novo loss-of-function (LoF) variants in *KMT2A* are associated with Wiedemann−Steiner Syndrome (WSS). WSS is characterized by hypertrichosis cubiti (hairy elbows), dysmorphic facial features, short stature, developmental delay and intellectual disability (ID).^5^

Since the initial association between WSS and *KMT2A*,^5^ additional patients with WSS and de novo LoF or missense variants in *KMT2A* have been reported^5–7^ (refer to Supplementary Table 1 for additional references). Although neurodevelopmental features and behavioural abnormalities were frequent, the lack of detailed neuropsychological assessments suggests the presence of these features may be underestimated and warrant further investigation.^7^ Whole-exome sequencing (WES) and whole-genome sequencing (WGS) studies in individuals with neurodevelopmental disorders (NDDs), including Autism Spectrum Disorder (ASD), have also revealed de novo *KMT2A* variants in 0.04−2.17% of individuals with NDDs^8–12^ (refer to Supplementary Table 1 for additional references). However, these studies that identified *KMT2A* variants in WSS and NDD cohorts were performed independently, and a comprehensive analysis of NDD and WSS phenotypes together in individuals with *KMT2A* variants has not been conducted. Here, we present the detailed clinical and genomic characterization of six unrelated individuals with de novo LoF or missense variants in *KMT2A*, and contextualize the findings with published literature.

## Results

### Clinical reports

#### Patient 1

A 10-year-old Caucasian male with a history of ASD, ID, attention deficit hyperactivity disorder (ADHD), hypotonia, growth and developmental delay was born to non-consanguineous parents of Ashkenazi Jewish ancestry, as a product of an in vitro fertilization twin pregnancy. He was delivered by Caesarian section at 34 weeks gestation, weighing 3 lb, 2 oz. He had poor growth with height and weight below the third percentile. A magnetic resonance imaging (MRI) of the brain at 2 years of age identified hypoplastic olfactory nerves and unusual configuration of the corpus callosum, showing a short dimension in anterior-posterior diameter and thinning of its body. His medical history is remarkable for delayed motor and speech milestones, hypotonia, bilateral cryptorchidism (surgically repaired), bilateral strabismus (surgically repaired) and constipation. He was diagnosed with ASD (age 5), ADHD (age 7), and ID (age 8). He takes Clonidine for ADHD and melatonin for trouble initiating sleep and frequent night awakenings.

**Fig. 1:**
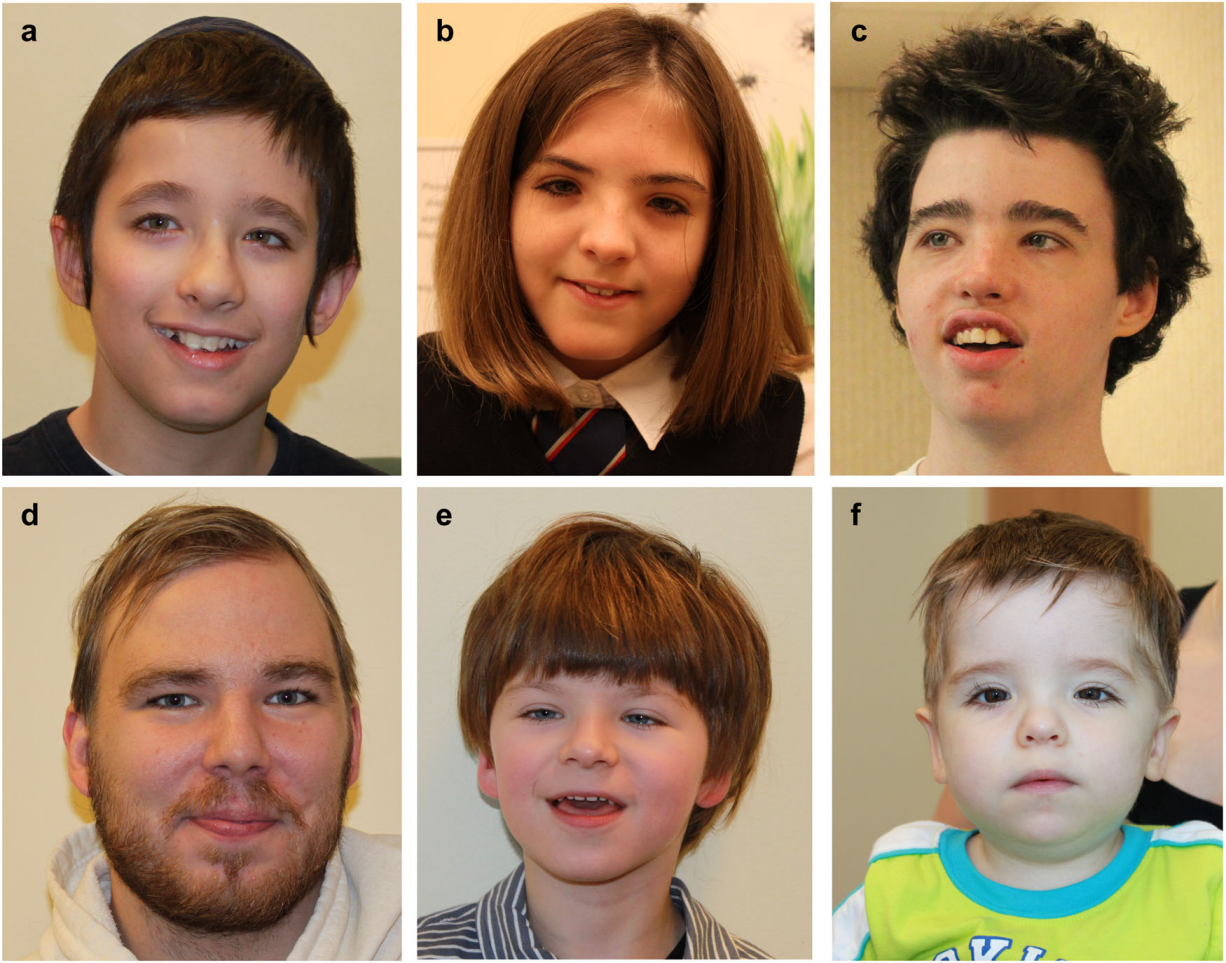
Facial features of six novel patients with de novo LoF or missense variants in *KMT2A*. **a** Patient 1 at 10 years; **b** patient 2 at 9 years; **c** patient 3 at 13 years; **d** Patient 4 at 25 years; **e** patient 5 at 5 years; **f** patient 6 at 1 year of age. Characteristic facial features noted in these patients include: thick and/or high arched eyebrows, long eyelashes, hypertelorism, downslanting palpebral fissures and a broad nasal tip. Written consent was obtained from all patients for open access publication of the photographs. LoF: loss-of-function

G-banded karyotype, fragile X testing and chromosome microarray (CMA) were normal. WGS identified a de novo *KMT2A* frameshift variant, c.10324delG (p.Ala3442Profs*17; Supplementary Fig. 1). He was then clinically assessed at age 10 and diagnosed with WSS on the basis of characteristic facial features (Fig. 1a), short stature, microcephaly, generalized hypertrichosis, and aforementioned history of growth and developmental delay, hypotonia, constipation, and strabismus (details in Supplementary Table 2).

As a part of this study, neurodevelopmental testing at 10 years, 3 months of age (Table 1) was consistent with his previous diagnoses of ID, ASD, and ADHD. This assessment also identified emotional dysregulation and extremely low language and adaptive skills, but relative strength in vocabulary skills. We observed many repetitive and restrictive behaviours (e.g. fixation with technology, buses, music). Of note, he struggles with transitions between settings and activities, becoming easily upset and oppositional. Despite overall difficulties with social-communication, he demonstrates some appropriate skills, such as making good eye contact, sustaining short conversations, and asking/offering information, particularly when the topic revolved around his area of interest.

**Table 1:** Summary of genetic results and neurodevelopmental profiles for six novel patients

	Patient 1	Patient 2	Patient 3	Patient 4	Patient 5	Patient 6
Sex	M	F	M	M	M	M
Technology used to identify *KMT2A* variant	WGS^a^	WES^b^	WGS^a^	WGS^a^	WES^b^	Targeted sequencing
*KMT2A* variant^c^	p.(Ala3442Profs*17)	p.(Ser2363Leufs*12)	p.(Val2057Tyrfs*18)	p.(Glu2566Lysfs*14)	p.(Leu2848Pro)	p.(Arg2699*)
Inheritance	De novo	De novo	De novo	De novo	De novo	De novo
Other variants	None	Maternally inherited 295 kb deletion chr4q31.3^d^ (chr4:151,378,576-151,673,967)	Maternally inherited *PAX1* p.(G166A)^d, e^	None	Maternally inherited *CHRNA4* c.(274-1 G > A)^d, f^	None
Age at Neurodevelopmental Assessment	10 y 3 m	12 y 2 m	13 y 1 m	25 y 3 m	5 y1m	5y 9 m
Intellectual Disability Diagnosis	+	+	+	−	+	+
IQ (percentile)	<1st	3rd	<1st	4th	<1st	<1st
Overall Adaptive Skills (percentile)	4th	1st	<1st	9th	<1st	<1st
Language Skills (OWLS-II^g^ percentile)	<1st	<1st	<1st	32nd	<1st	1st
Receptive Vocabulary (PPVT-4^h^ percentile)	16th	9th	1st	70th^i^	<1st	7th
Expressive Vocabulary (EVT-2^j^ percentile)	7th	5th	<1st	N/A^k^	<1st	1st
ASD Diagnosis	+	−	+	+	+	+
Rigidity/ Poor flexibility	+	+	+	+	+	+
Anxiety Symptoms	−	+^l^	+	−	−	+
Attention Concerns	+^m^	+^m^	+	−	+	+
Emotional Regulation Concerns (Dysregulation)	+	+	+	−^n^	+	+
Externalizing Behaviours	+	−	+	−	+	+

^a^Whole-genome sequencing

^b^Whole-exome sequencing

^c^*KMT2A* transcript used is NM_0011904.1

^d^Variant of unknown significance, not clinically significant

^e^*PAX1* transcript used is NM_006192

^f^*CHRNA4* transcript used is NM_000744.6

^g^Oral and Written Language Scales, Second Edition

^h^Peabody Picture and Vocabulary Test, Fourth Edition

^i^Based on previous assessment at 19 y

^j^Expressive Vocabulary Test, Second Edition

^k^EVT-2 not administered. Expressive language skills from OWLS-II

^l^Has diagnosis of Generalized Anxiety Disorder

^m^Has diagnosis of Attention Deficit Hyperactivity Disorder

^n^Behavior Rating Inventory of Executive Function not administered due to age. Information based on Antisocial Personality Problem Scale on the Adult Behavior Checklist as well as parent report

#### Patient 2

A 12-year-old Caucasian female with a history of ADHD, ID, growth and developmental delay, and hypotonia was born to non-consanguineous Caucasian parents at 36 weeks gestation with a birth weight of 5 lb, 9 oz. She had poor growth in infancy, with height and weight below the third percentile. Her medical history is remarkable for delayed motor milestones, a ventricular septal defect (which closed spontaneously), strabismus, hypotonia, constipation, recurrent upper respiratory tract infections, and Klippel−Feil anomaly. An MRI of the brain at 10 months of age identified mildly prominent cerebral spinal fluid spaces with age appropriate myelination. At 12 years of age she presented with episodes of rigidity and flexion of the arms with tremulous movements. An electroencephalography (EEG) was normal and the neurology team suspected the movements could represent self-stimulating behaviours. She was diagnosed with ADHD and ID (age 9) and generalized anxiety disorder (age 10). She also has obsessive compulsive traits (compulsive hand washing) and has received behavioural therapy throughout childhood to present.

**Fig. 2:**
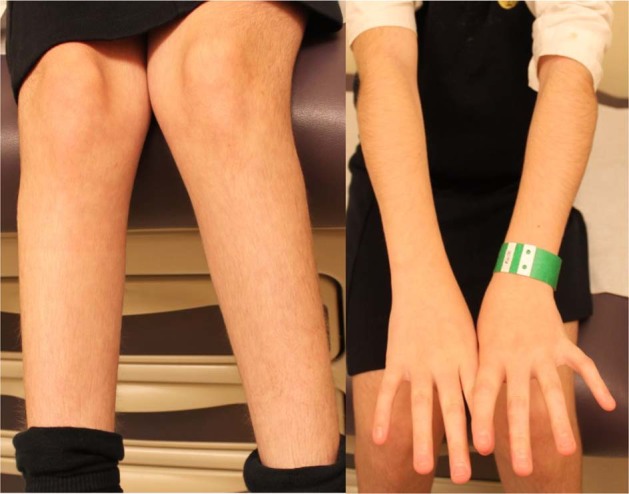
Hypertrichosis of the arms and legs of patient 2 at 9 years of age. Written consent was obtained from patient 2 for open access publication of the photographs

Clinical genetic assessments at 1 year 8 months of age included clinical CMA, fragile X testing, and metabolic screening. The latter two tests were normal; the microarray analysis identified a maternally inherited 295 kb deletion at chromosome 4q31.3 (chr4:151,378,576−151,673,967) that was not suspected to be clinically significant. After we identified a de novo *KMT2A* frameshift variant, c.7087_7090del (p.Ser2363Leufs*12; Supplementary Fig. 1) via WES, she was then clinically re-assessed at age 12 and diagnosed with WSS on the basis of characteristic facial features (Fig. 1b), generalized hypertrichosis (Fig. 2), and the history of growth and developmental delay, hypotonia, constipation, and strabismus (Supplementary Table 2).

As a part of this study, neurodevelopmental testing at 12 years, 2 months of age (Table 1) was consistent with the previous diagnosis of ID, ADHD and anxiety disorder. The assessment also identified emotional dysregulation and extremely low language and adaptive skills, but relative strength in vocabulary skills. Standardized testing for ASD revealed that she met criteria on the Autism Diagnostic Observation Schedule, Second Edition (ADOS-2), but did not meet criteria on the Autism Diagnostic Interview—Revised (ADI-R). Based on overall clinical impression, she did not receive a diagnosis of ASD. Nevertheless, a pattern of restricted and repetitive behaviours was identified on the ADOS-2, ADI-R and parent report. She struggles with changes to routine and becomes easily frustrated. Of note, clinical impressions were of a socially motivated girl, whose relative strength in vocabulary masks her areas of difficulty and she presents as having a higher level of comprehension.

#### Patient 3

A 13-year-old Caucasian male with a history of ASD, growth and developmental delay and hypotonia was born to non-consanguineous Caucasian parents at term by caesarean section with a birth weight of 6 lb, 14 oz. His mother was on citalopram during the pregnancy for treatment of depression. He was diagnosed with grade five vesicoureteral reflux in infancy with a dysplastic kidney on the left. He experienced absence seizures at 3 months of age and again at 9 years. An EEG at 9 months of age was normal. A computed tomography scan of the brain at three months showed delayed myelination. A brain MRI at two and a half years showed hypoplastic olfactory nerves, a Klippel−Feil anomaly, and incomplete myelin maturation in the inferior frontal lobes and temporal tips. Growth parameters were at or below the third percentile throughout childhood. He was hypotonic and there was a history of severe constipation. All of his developmental milestones were delayed. At about two and a half years, he was diagnosed with ASD. At about 11 years of age, he had symptoms of anxiety and Oppositional Defiant Disorder (ODD) although no official diagnosis was given. He was trialled on several anti-anxiety medications with no effect and is currently on resperidone. He has received extensive behavioural therapy from the time of ASD diagnosis to the present.

Clinical genetic assessments at 3 and 5 years of age did not identify a specific genetic diagnosis. Clinical CMA, fragile X testing, and metabolic testing were reported to be normal. We identified a de novo *KMT2A* frameshift variant, c.6169del (p.Val2057Tyrfs*18; Supplementary Fig. 1) via WGS. He was then clinically re-assessed at age 13 and diagnosed with WSS on the basis of characteristic facial features (Fig. 1c), microcephaly, hypertrichosis and history of growth and developmental delay, hypotonia, constipation, and ASD (details in Supplementary Table 2).

As a part of this study, neurodevelopmental testing at 13 years and 1 month of age (Table 1) confirmed previous diagnoses of ASD and ID. The assessment also identified emotional dysregulation and extremely low language/vocabulary and adaptive skills and symptoms of anxiety and ODD as reported previously. Concerns with attention were also endorsed by parents. Of note, he has significant difficulty with restricted and repetitive behaviours as identified by scores on the ADOS-2, as well as observations during the assessment. He struggled with transitions between settings and activities, becoming easily upset and requiring frequent breaks from work. Despite overall difficulties with social-communication, he demonstrates emerging skills, such as interest and engagement in social interactions as observed clinically and on multiple items on the ADOS-2.

#### Patient 4

A 25-year-old Caucasian male who was born to non-consanguineous Caucasian parents with a birth weight of 6 lb, 11 oz. has a history of short stature with growth consistently at the third percentile, hypotonia and delayed motor milestones. He was diagnosed with ASD (Asperger syndrome) at 10 years of age and had hyperlexia and symptoms of Tourette syndrome, specifically verbal tics.

Chromosomal microarray was performed on a research basis and the results were normal. After we identified a de novo *KMT2A* frameshift variant, c.7695_7696del (p.Glu2566Lysfs*14; Supplementary Fig. 1) via WGS, he was clinically re-assessed at age 25 and diagnosed with WSS on the basis of characteristic facial features (Fig. 1d), history of generalized hypertrichosis, and history of growth and developmental delay, cryptorchidism, hypotonia, and ASD (Supplementary Table 2).

Neurodevelopmental testing, as part of this study, at 25 years, 3 months of age (Table 1) confirmed previous diagnoses of ASD. He has developed good social-communication skills over the years, which contributed to an ADOS-2 score below clinical cut-off. However, based on overall clinical impressions he continues to meet criteria for ASD with notable difficulties being flexible in conversations, providing insight into others’ emotions, and understanding subtle nuances in social situations. Abnormality in speech rhythm, intonation and volume were also noted. This assessment identified borderline cognitive skills, low average adaptive skills, and average language and vocabulary skills. No concerns around anxiety, attention, aggression and emotional regulation were endorsed by parents or through clinical observation.

#### Patient 5

A 5-year-old Caucasian male with a history of ASD, growth and developmental delay, microcephaly, hypotonia, and esotropia was born at term to non-consanguineous Caucasian parents and had a birth weight of 6 lb, 7 oz. At 2 months of age he was hospitalized for investigation of nonepileptic paroxysmal events, with recurrent agitation, fist clenching, movement of arms and legs and screaming. Investigations including EEG and barium swallow were reported to be normal. At 3 months of age he began experiencing feeding difficulties with poor growth (weight below the third percentile). Due to ongoing feeding difficulties, a G-tube was inserted at 11 months. A brain MRI at 11 months identified cystic lesions in the pineal region and the pituitary fossa. Repeat MRI at 3 years also noted a dysplastic corpus callosum, hypoplastic optic nerves and a Klippel-Feil anomaly. His medical history is also remarkable for microcephaly, hypotonia, esotropia, constipation, bilateral orchidopexy and surgery for a tongue-tie release. All of his developmental milestones were delayed. He was subsequently diagnosed with ASD at 5 years of age and is on the waitlist for behavioural therapy.

Initial clinical genetics assessment at 8 months of age included clinical CMA, metabolic investigations and molecular testing for Prader−Willi syndrome and spinal muscular atrophy, which were all negative. At 19 months of age, a gene panel of 392 ID genes (University of Chicago) identified a maternally inherited variant in *CHRNA4* and not suspected to be clinically significant. To date, he does not have evidence of seizures. At three and a half years of age WES was clinically requested and identified a de novo missense variant in *KMT2A*, c.8543 T > C (p.Leu2848Pro). He was clinically re-assessed at 5 years of age and noted to have facial features characteristic for WSS (Fig. 1e), generalized hypertrichosis and the history described above (Supplementary Table 2).

Neurodevelopmental testing, as part of this study, was conducted at 5 years, 1 month of age (Table 1) and confirmed the previous diagnosis of ASD. Based on the ADOS-2, ADI-R, and clinical observations, he had most difficulty with flexibility, following another person’s lead, and sensory-seeking behaviour. Despite difficulties with areas of social-communication, he demonstrated motivation for social interaction and appropriate use of facial expressions. This assessment also identified extremely low cognitive, language and adaptive skills, leading to a diagnosis of ID. Of note, when his demands are not met, he exhibits aggressive and self-injurious behaviours as reported on the Child Behavior Checklist (CBCL) and ADI-R. We observed concerns around attention and scores on the CBCL were significantly elevated. Assessment of emotional regulation showed significantly elevated scores, indicating dysregulation.

#### Patient 6

A 5-year-old Caucasian male who was born to non-consanguineous Caucasian parents at term by Caesarian section with a birth weight of 7 lb, 11 oz. had poor growth in infancy, with height and weight below the third percentile. His medical history is remarkable for feeding difficulties with gastroesophageal reflux, a ventricular septal defect (which closed spontaneously), hypotonia, severe constipation (requiring hospitalization for bowel cleanout on several occasions) and recurrent urinary tract and upper respiratory infections. At 22 months of age he experienced seizures during the process of bowel cleanout. These were suspected to be related to hypoglycemia secondary to fasting. Investigations did not identify a metabolic aetiology for the hypoglycemia. An EEG identified abnormal epileptiform discharges. MRI identified a pineal cyst, craniocervical junction stenosis and a Klippel−Feil anomaly. He subsequently experienced several seizure-like episodes with eye-rolling and arm extension in association with intercurrent illnesses and stress related to medical procedures. A repeat EEG was reported to be normal. It was suspected that these episodes may be due to atypical vasovagal syncope. All of his motor milestones were delayed. A school behavioural assessment at 4 years of age noted concerns around non-compliance, physical aggression and tantrums, disruptive behaviours, and touching/taking other’s possessions. No diagnosis was given at this time, but extensive accommodations were implemented at school.

Initial clinical genetics assessment at 23 months of age included clinical CMA, and molecular testing for Russell−Silver syndrome and Smith−Lemli Opitz syndrome, which were all negative. He was re-assessed at 31 months of age and based on the observation of hypertrichosis (arms and back), dysmorphic facial features, failure to thrive and constipation, targeted testing of *KMT2A* was requested clinically, which identified a de novo nonsense variant, c.8095 C > T (p.Arg2699*). He was clinically re-assessed at 5 years of age as part of this study and noted to have facial features characteristic for WSS (Fig. 1f), generalized hypertrichosis and the history described above (see Supplementary Table 2).

His first neurodevelopmental assessment was conducted as part of this study at the age of 5 years, 9 months (Table 1), at which time he received a diagnosis of ASD and ID. He met criteria for ASD based on the ADOS-2, ADI-R and clinical judgement. He had most difficulty with repetitive interests, fixations with items, and transitions and changes in routine. He demonstrated shared enjoyment during social interactions, appropriate eye contact and facial expressions, and was able to point to objects of interest, indicating a relative strength in social-communication. This assessment also identified extremely low language and adaptive skills, but relative strength in receptive vocabulary skills. Of note, he demonstrates rigidity and poor flexibility in his behaviour and difficulty regulating his emotions, becoming easily upset and anxious when things are not done his way, which can lead to aggressive behaviours. Concerns around attention were significantly elevated based on the CBCL and clinical observations. He is currently on a waitlist for behavioural therapy.

### Nuclear family assessments

In all patients, assessment of cognitive functioning of parents (*N* = 11) and all siblings (*N* = 4) identified an IQ ranging from average to superior. Symptoms of anxiety were commonly endorsed by parents, although we did not perform formal anxiety diagnostic assessments. In both patients where the proband had a diagnosis of ADHD, one parent reported having attention difficulties in themselves. In one of these families a sibling also had a diagnosis of ADHD, although did not report current symptoms. In five of the six patients (except for patient 4), ASD traits were present in at least one parent, although we did not perform formal ASD diagnostic assessments. There were no ASD traits identified in any of the siblings and diagnostic testing for ASD was not warranted. No other obvious familial patterns impacting neurodevelopment were identified within or across the six patients.

### Meta-analysis of *KMT2A* variants

**Fig. 3:**
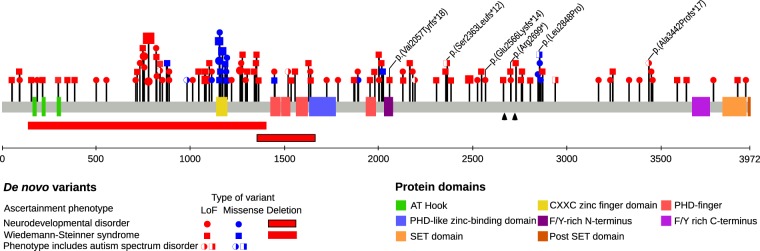
Distribution of de novo LoF, missense, and deletion variants in KMT2A from novel cases and individuals from meta-analysis. One hundred and three de novo LoF, 24 de novo missense, and two de novo deletion variants are plotted on the KMT2A protein (NM_001197104.1) with the protein coordinates below. The six new patients are shown with Human Genome Variation Society nomenclature. The two black arrows indicate the two taspase 1 cleavage sites that generate the N-terminal and C-terminal fragment. Size of the circle or square is proportional to the number of individuals with that specific KMT2A variant. LoF: loss-of-function

We conducted a meta-analysis to identify other *KMT2A* variants in individuals with WSS and/or NDDs. Including our six patients, we identified 127 individuals with WSS or NDD and de novo *KMT2A* LoF and missense variants (Fig. 3 and Supplementary Data). Using *KMT2A* variants from WES and WGS studies (Supplementary Table 1 and Supplementary Data), we found that individuals ascertained for NDDs were significantly enriched for de novo LoF and missense variants (*p* = 6.15 × 10^−4^) and de novo LoF variants alone (*p* = 1.92 × 10^−3^) when compared with controls, but not for de novo missense variants alone (*p* = 0.321) (Table 2). The lack of statistical significance for de novo missense variants might have been due to the decreased power from the limited number of control samples used to detect the smaller effect sizes of missense variants. Therefore, we also determined whether our observed numbers of de novo LoF and missense variants among individuals with NDD were greater than those expected based on a statistical model of de novo LoF and missense mutation rates for this gene, as determined previously.^13^ The observed numbers of de novo LoF variants (*p* = 5.96 × 10^−69^), de novo missense variants (*p* = 0.016), and both combined (*p* = 8.92 × 10^−41^) were all significantly increased in the NDD cohort, compared with the expected number of de novo LoF and/or missense variants derived from the mutation rate of *KMT2A*^13^ (Table 2).

**Table 2:** Evaluation of different variant classes in *KMT2A* in individuals with neurodevelopmental disorders (NDDs)

	Count (%)		*p* value	
	Individuals with NDDs	Control	vs. control^a^	vs. expected number of variants^b^
SNV/indel	(*n* = 15,070)	(*n* = 2299)		
De novo LoF	44 (0.29%)	0 (0%)	1.92 × 10^−3^	5.96 × 10^−69^
De novo missense	8 (0.05%)	0 (0%)	0.321	0.016
Combined	52 (0.35%)	0 (0%)	6.15 × 10^−4^	8.92 × 10^−41^
CNV	(*n* = 5815 ^c^)	(*n* = 46,652)		
Deletions	2	2	0.063	NA
Duplications	2	7	0.19	NA

^a^Fisher’s exact test, one-sided

^b^Binomial test. Expected number of variants for 15,070 individuals was determined using the de novo mutation rate model in Samocha et al.

^c^For deletions, *n* = 5815 and for duplications, *n* = 4681

To determine whether the de novo LoF and missense variants assort to certain regions of the KMT2A protein according to associated phenotypes, we examined the distribution of these variants. We found de novo LoF and missense variants to be dispersed throughout KMT2A and no obvious genotype−phenotype correlation (with respect to WSS, NDDs and ASD) (Fig. 3). There is a cluster of 13 de novo missense variants in the CXXC zinc finger binding domain, of which p.(Arg1154Trp), p.(Cys1155Tyr), and p.(Gly1168Asp) are recurrent variants found at similar frequencies in individuals with WSS or NDDs (Fig. 3 and Supplementary Table 3). With regards to copy number variants (CNVs), deletions and duplications (*p* = 0.063 and 0.19, respectively) in *KMT2A* were not significantly enriched compared with controls (Table 2, Supplementary Fig. 2).

**Fig. 4:**
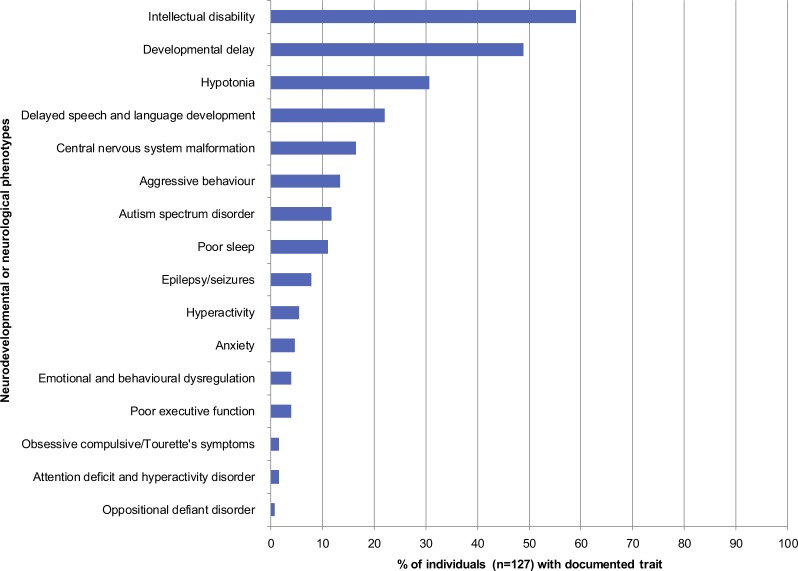
Spectrum of reported neurodevelopmental and neurological phenotypes of 127 individuals with de novo LoF or missense variants in *KMT2A*. Frequencies of neurodevelopmental and neurological phenotypes reported in publications are shown. Note that absence of phenotype from a publication either means the phenotype was not assessed or there was a negative finding, which we could not distinguish in most publications. LoF: loss-of-function

To dissect the neurodevelopmental spectrum of de novo *KMT2A* variants, we examined the reported phenotypes of the 127 individuals with de novo LoF or missense variants in *KMT2A*. The top five reported phenotypes were ID (59.1%), developmental delay (48.8%), hypotonia (30.7%), delayed speech and language development (22.0%), and malformations in central nervous system (16.5%) (Fig. 4). ASD was described in 11.8% of individuals (Fig. 4). Of note, detailed phenotyping was not available for the majority of individuals. ADHD, ODD, Tourette Syndrome, and obsessive compulsive symptoms were observed in our patients and have not been previously reported in individuals with de novo *KMT2A* variants.

## Discussion

We describe six individuals with WSS and a characteristic spectrum of NDDs with de novo LoF or missense variants in *KMT2A*. We did not identify other clinically significant variants that contributed to the observed NDD phenotypes, suggesting that heterozygous mutations in *KMT2A* alone is sufficient to explain the observed NDD phenotypes.^14^ In line with recent reports that identified *KMT2A* as a candidate gene for ASD^11,15^ and NDDs,^8,12^ our findings support *KMT2A* as a high confidence NDD susceptibility gene similar to *ARID1B* and *KMT2C*. De novo LoF and missense variants in *KMT2A* are highly penetrant and can explain the neurodevelopmental and phenotypic features of WSS. This is supported by a high constraint for LoF and missense mutations in *KMT2A* (the probability of LoF intolerance score is 1.00 and the z-score for missense mutations is 6.64 ^16^). Collectively, our findings can contribute to increased molecular diagnostic yields for NDDs and ASD.^17,18^

Prior to identifying the *KMT2A* variants, patients 1−5 had a diagnosis of NDD (three were ascertained for ASD), but no identified syndrome. Only patient 6 had a clinical diagnosis of WSS, which was confirmed molecularly. After identification of *KMT2A* variants and clinical reassessment, patients 1−5 met the criteria for WSS, suggesting that WSS is often under-recognized. With the increased utilization of WES/WGS in the clinical and research settings, we anticipate that the diagnostic rate of WSS will increase.^7,19^

Prior to this study, the NDD phenotype in individuals with *KMT2A* variants had not been systematically assessed using specific neuropsychological tests. A recent paper by Baer et al. highlighted the need for more in-depth NDD phenotyping.^7^ Our deep phenotyping analysis delineated a particular ASD subtype in association with *KMT2A* variants. Poor language skills/delayed speech has been previously reported in individuals with *KMT2A* variants. The six individuals in our study were able to speak in short phrases or full sentences, giving the impression of higher abilities than were found on direct assessments. Specifically, vocabulary, higher-order language skills and cognitive functioning of five of our patients were delayed. The emerging ASD subtype included social motivation to interact with others (despite social-communication difficulties), paired with rigid, repetitive and inflexible behaviours (e.g. difficulty switching from preferred topics), emotional dysregulation and externalizing behaviours that limited their ability to sustain social interactions. Although some ASD features were present in at least one parent of the four out of five patients with ASD, autistic traits have been shown to exist in a continuous distribution in the general population with family members of individuals with ASD having a greater likelihood of exhibiting subclinical autistic traits.^20–22^ We did not find clinically significant inherited rare variants that contributed to ASD risk in our patients. This does not rule out additional genetic factors that may be contributing to ASD risk in these families,^14^ such as common variants, rare variants with smaller effect sizes, noncoding variants, other structural variants, or variants whose contribution is still unknown. Nevertheless, our findings suggest that de novo LoF and missense variants in *KMT2A* are associated with WSS, a syndromic form of NDD with increased risk of a particular ASD subtype, providing further delineation of the neurodevelopmental phenotype associated with WSS.

Functional studies in conditional knockout mice deficient in *Kmt2a* in various brain regions also support a role for *KMT2A* in neurodevelopment. *Kmt2a* is known to regulate genes related to neurogenesis^23^ and is required for neurogenesis in neural stem cells in the subventricular zone.^3^ Moreover, neurons from *Camk-Cre*^*+*^ *Kmt2a*^*2lox/2lox*^ mice exhibit significant changes in H3K4me3 and gene expression levels in orthologues of neuropsychiatric genes,^24^ and significant changes in expression of genes related to the synapse, axon, membrane,^24^ cognition and mood.^25^ These findings are reflected in the behavioural phenotypes in the mutant mice: ablation of *Kmt2a* in the forebrain resulted in increased anxiety, hyperactivity, spatial working memory deficits, and impaired social behaviours,^24,25^ whereas ablation of *Kmt2a* in the striatum, it resulted in increased anxiety.^25^ Some of these phenotypes (i.e. impaired social behaviours, anxiety, and hyperactivity) are also observed in individuals carrying de novo *KMT2A* variants.

Skeletal anomalies are a known feature of WSS with a recent study documenting Klippel−Feil anomaly in five patients with WSS and de novo *KMT2A* variants.^7^ Among our six patients, four have Klippel−Feil anomaly. Skeletal phenotypes have also been observed in *Kmt2a*^*+/*−^ mice, including fusion of cervical vertebrae.^26^ In mouse embryonic fibroblasts, *Kmt2a* regulates the expression and H3K4 trimethylation of *Gdf6* (a gene involved in bone morphogenesis that causes Klippel–Feil Syndrome (KFS)), *Pax1 (*KFS candidate gene^27^), and *Pax9* (a functionally relevant gene in vertebrate segmentation^28^). WES or WGS in patients 2, 3, and 5 did not identify variants in genes known to be associated with KFS (*GDF6*, *MEOX1*, *GDF3*, *MYO18B*), except for a maternally inherited missense variant in *PAX1* in patient 3 (Table 1). These findings suggest that skeletal anomalies may be a more common feature of WSS than previously reported, due to dysregulation of genes involved in bone morphogenesis, as a result of *KMT2A* dysregulation.

In conclusion, we have demonstrated that individuals with de novo LoF or missense variants in *KMT2A* likely have a clinically unrecognized diagnosis of WSS, rather than non-syndromic NDD. Our findings further characterize the neurodevelopmental phenotype associated with WSS, revealing a characteristic subtype of ASD. We recommend that any individual with a de novo *KMT2A* LoF or missense variant undergo both clinical genetics and neurodevelopmental assessments.

## Methods

### Study participants

Participants for the clinical report series were recruited at the Hospital for Sick Children. Four of the six patients were identified as part of an ongoing research study investigating the genetic aetiology of ASD and neurodevelopmental conditions. The remaining two patients were identified through the Division of Clinical and Metabolic Genetics at the Hospital for Sick Children. Written informed consent approved by the Research Ethics Board at The Hospital for Sick Children was obtained for the study. The authors affirm that human research participants provided informed consent for open access publication of the images in figures. All six families underwent a clinical assessment by a geneticist (R.W.) and a neurodevelopmental assessment supervised by a psychologist (I.D. and A.R.). The clinical genetics assessment involved a review of the proband’s medical and family histories and a physical dysmorphology examination. The neurodevelopmental assessment involved psychological assessment of the proband and available first-degree relatives as described below.

### Neurodevelopmental phenotype

Probands were assessed using validated, standardized multi-method approaches and assessments. Where available, and still valid (i.e. completed within past 2 years), the scores from assessments completed at other clinics were used to decrease test burden. Direct (individualized assessments, interviews) and indirect (questionnaires) assessments used to evaluate ASD, cognitive functioning, language skills (including receptive and expressive), vocabulary (receptive and expressive), executive functioning skills, behavioural and emotional concerns (specifically anxiety/depressive symptoms, attention concerns, obsessive compulsive tendencies, emotional dysregulation, externalizing behaviours), and adaptive functioning are described in supplementary methods. The available nuclear family members of each patient were assessed using validated, standardized multi-method approaches to evaluate any familial patterns or impacts on neurodevelopment. Areas evaluated included cognitive functioning, vocabulary (receptive and expressive), ASD traits, and behavioural and emotional concerns.

### WGS analysis for patients 1, 3, and 4

Through the Autism Speaks MSSNG portal,^11^ we screened WGS data of 2414 ASD parent−child trios for rare (<1% in population control databases) de novo LoF and damaging missense (predicted damaging in five of seven prediction algorithms as previously described^29^) variants in *KMT2A* (NM_0011904.1). We selected those with a genotype quality score ≥99 and with 30–70% of reads supporting the alternative allele, for validation using Sanger sequencing. Rare SNVs and indels were downloaded from MSSNG and annotated as previously described.^11^ We used a previously recommended workflow^30^ to detect CNVs larger than 1 kb from WGS data, using read depth-based algorithms: ERDS and CNV-nator. We calculated an internal frequency filter using the ERDS and CNVnator calls of the 4828 unaffected parents in MSSNG and prioritized rare (<1% allele frequency in unaffected parents) CNVs.

### WES analysis for patient 2

We sequenced DNA from whole blood of patient 2 using Illumina HiSeq2500, after exome enrichment with SureSelect V5 Capture Kit (Aligent). We used Burrows−Wheeler Aligner v.0.5.9 ^31^ to align WES reads, Genome Analysis Toolkit Haplotype caller v.1.1-28 ^32^ to call variants. We selected variants relevant to NDDs (discussed below) for Sanger sequencing confirmation using DNA extracted from the blood of patient 2 and her parents.

### Identifying clinically significant variants

To identify clinically significant variants in patients 1−4, we prioritized rare (<1% allele frequency in population controls) LoF or missense variants predicted to be damaging by at least five of seven criteria, as previously described,^29^ and variants reported in ClinVar^33^ and the Human Gene Variant Database.^34^ We also prioritized rare CNVs (<1% allele frequency in population controls and <1% allele frequency in parents from the MSSNG database), including those overlapping syndromic regions in ClinGen Genome Dosage Sensitivity Map^35^ databases, which contains expert-curated CNVs involved in developmental disorders. Genes affected by such variants were compared with ASD candidate genes,^11,12,36–38^ candidate genes for NDDs,^12^ and genes implicated in neurodevelopmental or behavioural phenotypes according to the Human and Mouse Phenotype Onotologies.^39,40^ Additionally, we considered the mode of inheritance from the Online Mendelian Inheritance in Man and Clinical Genomics Database^41^ and the segregation in the family. Then we classified the variants as clinically significant (pathogenic or likely pathogenic) or of unknown significance, based on the American College of Medical Genetics and Genomics (ACMG) guidelines.^42,43^

### Meta-analysis of *KMT2A* variants

We conducted a literature search on the PubMed database for articles published between January 2011 and August 2018, to gather additional data from individuals with WSS or NDDs and variants in *KMT2A*. Search terms were: *KMT2A*, *MLL*, WSS, Wiedemann−Steiner Syndrome, ID, autism spectrum disorder, developmental delay, epilepsy, ADHD, OCD, schizophrenia, bipolar disorder, whole exome sequencing, and whole genome sequencing. We found 37 publications that identified *KMT2A* variants in individuals with WSS or NDDs (Supplementary Table 1).

To determine whether there was an enrichment of de novo *KMT2A* variants in NDD patients, we used WGS data from 250 Dutch parent−offspring trios^44^ and WES data from 138 parent−offspring trios^45–48^ and 1911 unaffected siblings^46^ (Supplementary Table 1) as controls. The total number of control trios was 2299.

We also screened a microarray database of 4681 ASD^37^ and 15,812 control individuals (unpublished data; and see Zarrei et al.^49^ for details about published controls) for CNVs that overlapped *KMT2A*. We considered rare CNVs (≤0.1% allele frequency in controls) that overlapped *KMT2A* and were called by two algorithms within the same microarray platform and validated these with Taq-Man Copy Number Assays. We also searched the Database of Genomic Variants^50^ for additional CNVs impacting *KMT2A* in controls.

### Reporting Summary

Further information on experimental design is available in the Nature Research Reporting Summary linked to this article.

## Supplementary information

Supplementary Information

Reporting Summary

Dataset 1

## Data Availability

WGS data can be accessed through the Autism Speaks MSSNG database (for access, see http://www.mss.ng/researchers). WGS data for patients 1, 3, and 4 and WES data for patient 2 are deposited in the European Genome-phenome Archive (www.ebi.ac.uk/ega/) under accession number EGSA00001003521. CNV control datasets used for the analyses described in this manuscript were obtained from dbGaP at https://www-ncbi-nlm-nih-gov.myaccess.library.utoronto.ca/projects/gap/cgi517bin/study.cgi?study_id=phs000092.v1.p1 through dbGaP accession number phs000092.v1.p1. Datasets supporting the conclusions of this article are included within the article, supplementary tables, and supplementary data. Other data that support the findings of this study are available from the corresponding author on reasonable request.

## Supplementary information

**Supplementary information** accompanies the paper on the *npj Genomic Medicine* website (10.1038/s41525-019-0083-x).
