# Supplementary material for: Expanding the neurodevelopmental phenotypes of individuals with de novo KMT2A variants
Source: NPJ Genom Med. 2019 Apr 26;4:9. doi: 10.1038/s41525-019-0083-x (PMC6486600; doi:10.1038/s41525-019-0083-x)
Supplement: Supplementary file 1 — Supplementary Information [file 41525_2019_83_MOESM1_ESM.docx]

**Supplementary Methods**

**Neurodevelopmental phenotype:**

Cognitive functioning and language skills were assessed through direct measures. Cognitive function (IQ) was assessed using one of the following age and ability dependent standardized tests: Wechsler Abbreviated Scale of Intelligence, Second Edition, Wechsler Preschool and Primary Scale of Intelligence, Fourth Edition, or Stanford-Binet Intelligence Scales, Fifth Edition. Language skills were assessed by the Listening Comprehension and Oral Expression subtests on the Oral and Written Language Scales, Second Edition. Receptive and expressive vocabulary were assessed using the Expressive Vocabulary Test, Second Edition and the Peabody Picture Vocabulary Test, Fourth Edition. Assessment for autism spectrum disorder was conducted using the Autism Diagnostic Observation Schedule, Second Edition, Autism Diagnostic Interview – Revised, Social Responsiveness Scale, Second Edition and Social Communication Questionnaire, Lifetime Version. These measures were also used to affirm observations of rigidity and poor flexibility. Behavioural, emotional, adaptive, and executive functioning skills were assessed using age-appropriate indirect measures. Anxiety symptoms were assessed using the Spence Children’s Anxiety Scale, Revised Child Anxiety and Depression Scale or Beck Anxiety Inventory. Attention concerns were assessed using the Strengths and Weaknesses of Attention Deficit/Hyperactivity Symptoms and Normal Behaviour rating scale and the Child Behavior Checklist (CBCL)/ Adult Behavior Checklist (ABCL). Emotional regulation concerns were assessed using either the Emotion Regulation Index of the Behavior Rating Inventory of Executive Function or the Antisocial Personality Problems scale of the ABCL. Externalizing behaviours were assessed using the CBCL/ ABCL. Obsessive compulsive tendencies were assessed using the Toronto Obsessive Compulsive Scale. Depressive symptoms were assessed using either the Children’s Depression Inventory, Second Edition or the Beck Depression Inventory, Second Edition. Adaptive skills were assessed using the Adaptive Behavior Assessment System, Second Edition.

**Supplementary Table 1: Papers used in meta-analysis.**

| Author | PMID | Technologies^1^ | Disorder^2^ | Number of probands with *KMT2A* mutations | Total number of probands | NDD statistics | |
| --- | --- | --- | --- | --- | --- | --- | --- |
| Jones, *et al*. (2012) | 22795537 | WES | WSS | 5 | 6 |  | |
| Mendelsohn, *et al*. (2014) | 24818805 | WES | WSS | 1 | 1 |  | |
| Strom, *et al*. (2014) | 24886118 | WES | WSS | 2 | 2 |  | |
| Cavel, *et al*. (2015) | 26544196 | WES, CGH array | WSS | 1 | 1 |  | |
| Steel, *et al*. (2015) | 26690532 | WES | WSS | 1 | 1 |  | |
| Dunkerton, *et al*. (2015) | 25929198 | WES, SNP microarray | WSS | 2 | 2 |  | |
| Yuan, *et al.* (2015) | 25574841 | WES | WSS | 1 (WSS) | 32 with CdLS |  | |
| Bramswig, *et al.* (2015) | 25724810 | WES | WSS | 1 (WSS) | 10 with tentative diagnosis of CSS^3^ or NCBRS^4^ | |  |
| Stellacci, *et al*. (2016) | 27320412 | WES | WSS | 1 | 1 |  | |
| Ko, *et al*. (2016) | 27777327 | WES, CMA, karotyping | WSS | 2 | 2 |  | |
| Miyake, *et al.* (2016) | 25810209 | WES | WSS | 6 | 6 |  | |
| Sun, *et al*. (2017) | 27759909 | WES, targeted capture sequencing | WSS | 2 | 2 |  | |
| Aggarwal, *et al.* (2017) | 28359930 | WES | WSS | 1 | 1 |  | |
| Enokizono, *et al.* (2017) | 28815892 | WES | WSS | 1 | 1 |  | |
| Bogaert, *et al.* (2017) | 28623346 | WES | WSS | 1 | 2 |  | |
| Parenti, *et al*. (2017) | 28120103 | targeted sequencing, WES | patients with CdLS features | 1 | 7 |  | |
| Baer, *et al*. (2018) | 29574747 | targeted, high-throughput or exome sequencing | WSS | 33 | 33 |  | |
| Stoyle, *et al.* (2018) | 30159147 | NGS-based ID panel | WSS | 1 | 1 |  | |
| Li, *et al*. (2018) | 30305169 | targeted sequencing/ WES | WSS | 14 | 14 |  | |
| Martinez, *et al*. (2016) | 27620904 | targeted sequencing of ID gene panel | ID | 2 | 92 |  | |
| Lebrun, *et al.* (2018) | 29203834 | NGS | ID | 41 | 200 |  | |
| Nambot, *et al.* (2018) | 29095811 | WES | ID + congenital anomalies | 3 | 416 | Y | |
| Lelieveld, *et al*. (2016) | 27479843 | WES | ID | 5 | 820 | Y | |
| Gilissen, *et al.* (2014) | 24896178 | WES | ID | 0 | 50 | Y | |
| de Ligt, *et al*. (2012) | 23033978 | WES | ID | 0 | 50^5^ | Y | |
| Rauch, *et al.* (2012) | 23020937 | WES | ID | 0 | 51 | Y | |
|  |  |  |  |  |  |  | |
| Author | **PMID** | **Technologies^1^** | **Disorder^2^** | **Number of probands with *KMT2A* mutations** | **Total number of probands** | **NDD statistics** | |
| Vissers, *et al.* (2010) | 21076407 | WES | ID | 0 | 10 | Y | |
| McRae, *et al*. | 28135719 | WES | DD/ID | 31^6^ | 4293 | Y | |
| Fitzgerald, *et al*. (2015) | 25533962 | WES | DD/ID | 1 | 1133^7^ | Y (denomina-tor no) | |
| Farwell, *et al*. (2015) | 25356970 | WES | ID/DD | 4 | 288^8^ | Y | |
| Bowling, *et al.* (2017) | 28554332 | WES/WGS | DD/ID | 0 | 309 | Y | |
| Lee, *et al.* (2014) | 25326637 | WES | DD | 2^9^ | 408^9^ | Y | |
| Hamdan, *et al.* (2017) | 29100083 | WGS | Develop-mental and epileptic encephalo-pathy | 0 | 197 | Y | |
| Halvardson, *et al*. (2016) | 27334371 | WES | ID and epilepsy | 0 | 39 | Y | |
| EuroEPINOMICS-RES Consortium, Epilepsy Phenome/Genome Project, and Epi4K Consortium. (2014) | 25262651 | WES | Epileptic encephalo-pathy | 1 | 356 | Y | |
| Heilberg, *et al.* (2016) | 26795593 | WES | Epilepsy | 3 | 254 | Y | |
| Ostrander, *et al.* (2018) | 30109124 | WGS | Early infantile epileptic encephalo-pathy | 0 | 14 | Y | |
| Yuen, *et al.* (2017) | 28263302 | WGS | ASD | 2 | 1630 |  | |
| MSSNG database (https://mssng.bioteam.net) | NA | WGS | ASD | 3^10^ | 2414^10^ | Y | |
| Turner, *et al*. (2016) | 26749308 | WGS | ASD | 1 | 53^11^ |  | |
| Krumm, *et al.* (2015) | 25961944 | WES | ASD | 3 | 2377^11^ |  | |
| Iossifov, *et al*. (2014) | 25363768 | WES | ASD | 1 | 2508 | Y | |
| De Rubies, *et al.* (2014) | 25363760 | WES | ASD | 2 | 1477 | Y | |
| McCarthy, *et al.* (2014) | 24776741 | WES | SCZ | 0 | 57 | Y | |
| Gulsuner, *et al.* (2013) | 23911319 | WES | SCZ | 0 | 105 | Y | |
| Xu, *et al.* (2012) | 23042115 | WES | SCZ | 0 | 231 | Y | |
| Xu, *et al.* (2011) | 21822266 | WES | SCZ | 0 | 53 | Y | |
| Fromer, *et al*. (2014) | 24463507 | WES | SCZ | 0 | 617 | Y | |
| Guipponi, *et al*. (2014) | 25420024 | WES | SCZ | 0 | 53 | Y | |
| Popp, *et al*. (2017) | 29158550 | Exome pool-seq | NDD | 1 | 96 |  | |
| Kataoka, *et al*. (2016) | 27217147 | WES | Bipolar disorder | 0 | 79 |  | |
| Iossifov, *et al*. (2014) | 25363768 | WES | Control | 0 | 1911 | Y | |
| Genome of Netherlands Consortium | 24974849 | WGS | Control | 0 | 250 | Y | |
| Author | **PMID** | **Technologies^1^** | **Disorder^2^** | **Number of probands with *KMT2A* mutations** | **Total number of probands** | **NDD statistics** | |
| Rauch, *et al.* (2012) | 23020937 | WES | Control | 0 | 20 | Y | |
| Gulsuner, *et al.* (2013) | 23911319 | WES | Control | 0 | 84 | Y | |
| Xu, *et al.* (2012) | 23042115 | WES | Control | 0 | 34 | Y | |
| Krumm, *et al.* (2015) | 25961944 | WES | Control | 1 | 2377^11^ |  | |

^1^WES = whole-exome sequencing, CGH = comparative genomic hybridization, CMA = chromosomal microarray, NGS = next generation sequencing, WGS = whole-genome sequencing

^2^WSS = Wiedemann-Steiner Syndrome, CdLS = Cornelia de Lange Syndrome, ID = Intellectual disability, ASD = Autism spectrum disorder, SCZ = Schizophrenia

^3^CSS = Coffin-Siris Syndrome

^4^NCBRS = Nicolaides-Baraitser Syndrome

^5^50 samples overlap between Gilissen, *et al.* and de Ligt, *et al.* only unique samples are reported for both studies

^6^The same *de novo KMT2A* mutations in four samples were reported in Fitzgerald, *et al.* and McRae, *et al.* papers and has only been counted in McRae, *et al.*

^7^Samples are a subset of McRae, et al. Only total trios from McRae, *et al.* was used

^8^Number of trios with ID and/or DD is not known. Of the 500 probands sequenced in Farwell *et al.*, 322 have ID and/or DD and of the 500, 288 are trios.

^9^Overestimation of number of trios with DD. 410 trios were sequenced in Lee *et al.*, of which 51% has DD, 20% with DD + hypotonia, 18% with DD + epilepsy/seizures, 24% with DD + dysmorphic features, 11% with DD + autism (not mutually exclusive categories). Two trios with *de novo KMT2A* variants were removed because they were already published in Strom, *et al.* (2014)

^10^Samples overlap between Yuen, *et al.* 2017 and Version 5 of MSSNG database, only number from version 5 of the MSSNG database were used

^11^Samples overlap with Iossifov *et al.* Only total trios from Iossifov *et al.* was used

**Supplementary Table 2: Clinical features of 6 novel patients**

| Patient ID | | Patient 1 | Patient 2 | Patient 3 | Patient 4 | Patient 5 | Patient 6 |
| --- | --- | --- | --- | --- | --- | --- | --- |
| Sex | | M | F | M | M | M | M |
| Age at deep phenotyping | | 10y | 12y 2m | 13y | 25y | 5y 1m | 5yr 9m |
| **Clinical genetic assessment** | |  |  |  |  |  |  |
| Facial features | |  |  |  |  |  |  |
|  | Microcephaly | + | - | + | - | + | - |
|  | Thick hair | + | + | + | - | + | + |
|  | Eyebrow lateral flare | - | + | + | + | + | + |
|  | Thick eyebrow | + | + | + | + | - | + |
|  | Long eyelashes | + | + | + | slightly long; thick | + | + |
|  | Long palpebral fissures | + | + | + | - | + | + |
|  | Downslanted palpebral fissure | + | + | - | + | + | + |
|  | Vertically narrow palpebral fissure | + | + | + | + | + | + |
|  | Short palpebral fissure | - | - | NA | - | - | - |
|  | Hypertelorism | + | + | NA | + | + | + |
|  | Epicanthus | - | - | + | - | - | - |
|  | Ectropion of lateral third of lower eyelid | + (mild) | - | - | - | - | + |
|  | Ptosis | - | + | - | - | + | - |
|  | Strabismus | + | + | NA | - | + | - |
|  | Short columella | - | - | + | - | + | + |
|  | Wide nasal bridge | - | + | + | + | - | + |
|  | Broad nasal tip | + | - | + | + | + | + |
|  | Prominent forehead | + (slight) | - | - | + | + | + |
|  | High arched eyebrows | + | + | + | - | - | + |
|  | Prominent ear | + Slight (left>right) | - | + | + (slightly) | + | - |
|  | Depressed nasal tip | - | - | - | - | - | - |
|  | Auricular deformity | + | - | + | - | + | - |
|  | Abnormal dentition | + (dental crowding) | early eruption of teeth | - | + (crowded) | early eruption of teeth | early dentition |
|  | Hypodontia | - | - | - | NA | - | - |
|  | High-arched palate | + | + | NA | + | + | + |
|  | Micrognathia | + | - | + | + | - | + |
|  | Cupid's bow, exaggerated | + (slight) | - | - | - | - | + |
|  | Thin upper vermillion border | + | + | + | + (slight) | + | + |
|  | Low posterior hairline | + | + | + | - | +, In centre only (v-shaped) | + |
| Skeletal abnormality | |  |  |  |  |  |  |
|  | Broad first digits | + | + | + | + | + (slight) | + |
|  | Tapering fingers | - | - | - | + (slight) | - | + |
|  | Long hallux | - (long 2nd toe) | + | NA | + (slight) | - | + |
|  | 2-3 toe syndactyly | + | + | NA | + | + | + |
|  | Small hand ( < -2SD) | + | - | - | + | + | + |
|  | Small foot ( < -2SD) | + | - | NA | - | + | + |
|  | Puffy hand | + (slight) | - | - | - | - | + (in infancy) |
|  | Puffy foot | - | - | - | - | - | - |
|  | Short fifth finger | - | - | - | - | - | + |
|  | Clinodactyly of fifth finger | + | - | - | + | + | + |
|  | Contracture of the DIP joint | - | NA | - | + | - | + |
|  | Spine/rib abnormality | NA | + (Klippel Feil Syndrome) | + (Klippel Feil Syndrome) | NA | +, fusion of C2-C3 bodies | + (Klippel Feil, occipitocervical junction stenosis) |
|  | Scoliosis | NA | - | - | - | - | - |
|  | Sacral dimple | NA | - | + | - | + | - |
|  | Hip joint dislocation | - | - | - | - | - | + |
|  | Advanced bone age | - | + | - | NA | NA | - |
|  | Joint laxity | - | - | + | NA | - | + |
| Skin features | |  |  |  |  |  |  |
|  | Generalized hirsutism | + | + | + | + | - | + |
|  | Hypertrichosis, cubiti | + | + | + | + | - | + |
|  | Hypertrichosis, back | + | + | NA | + | + | + |
|  | Hypertrichosis, lower limbs | + | + | + | + | + | + |
|  | Abnormal dermatoglyphics | + | + | NA | NA | NA | NA |
|  | Prominent digit pads | + | + | + | + | + | + |
| Neurological abnormalities | |  |  |  |  |  |  |
|  | Developmental delay | + | + | + | + | + | + |
|  | Hypotonia | + | + | + | + | + | + |
|  | Hypotonia in infancy | + | + | + | + | +, improved | + |
|  | Seizure | - | + | + | - | - | - |
|  | Poor sleep | + | + | - | + (disrupted sleep in childhood) | NA | + |
|  | Brain atrophy | - | - | - | NA | - | - |
|  | CNS malformation | +, Corpus callosum short dimension in SP diameter and thinning of body; small/hypoplastic olfactory nerves | - | + (incomplete myelin maturation and hypoplastic olfactory nerves) | NA | +, pineal and pituitary cysts, dysplastic corpus callosum and fornices, delayed myelination, hypoplastic optic nerves and optic chiasm | + (pineal cyst) |
| Growth problems | |  |  |  |  |  |  |
|  | Short stature (<-2SD) | + | - | + (during childhood) | + | - | + |
|  | Intrauterine growth retardation | + | - | - | - | - | - |
|  | Postnatal growth retardation | + | + | + | + | - | + |
|  | Slim and muscular build | + | - | slim but lanky | - | + | + |
| Internal organ problems | |  |  |  |  |  |  |
|  | Cardiovascular abnormality | - | + (VSD, resolved) | - | - | - | + (VSD, resolved) |
|  | Umbilical hernia | - | - | - | - | - | - |
|  | Constipation | + | + | + | - | + | + |
|  | Feeding difficulties | + | + | - | - | + | + |
|  | Nasogastric or percutaneous endoscopic gastrostomy | - | - | - | - | + | - |
|  | Kidney/ureter malformation | NA | NA | + | NA | - | - |
|  | Cryptorchidism | + | NA | - | + (Unilateral) | + | - |
|  | Deafness | - | - | - | - | - | - |
|  | Premature thelarche | NA | + | NA | NA | NA | NA |
| **Neurodevelopmental assessment** | |  |  |  |  |  |  |
| Cognitive functioning (IQ)^1^ | |  |  |  |  |  |  |
|  | WASI-II | N/A | 3rd %ile | N/A | 4th %ile | N/A | N/A |
|  | WPPSI-IV | N/A | N/A | N/A | N/A | N/A | <1st %ile |
|  | SB-5 | <1st %ile | N/A | <1st %ile | N/A | <1st %ile | N/A |
| Adaptive skills | |  |  |  |  |  |  |
|  | ABAS-II^2^ | 4th %ile | 1st %ile | <1st %ile | 9th %ile | <1st %ile | <1st %ile |
| Language skills & vocabulary^3^ | |  |  |  |  |  |  |
|  | OWLS-II | 1st %ile | <1st %ile | <1st %ile | 32nd %ile | <1st %ile | 1st %ile |
|  | PPVT-4 | 16th %ile | 9th %ile | 1st %ile | 70th %ile | <1st %ile | 7th %ile |
|  | EVT-2 | 7th %ile | 5th %ile | <1st %ile | N/A | <1st %ile | 1st %ile |
| Autism assessment^4^ | | ASD diagnosis | No ASD diagnosis | ASD diagnosis | ASD diagnosis | ASD diagnosis | ASD diagnosis |
|  | ADOS-2 | Met criteria for ASD | Met criteria for ASD | Met criteria for ASD | Did not meet criteria for ASD | Met criteria for ASD | Met criteria for ASD |
|  | ADI-R | Not completed | Did not meet criteria for ASD | Met criteria for ASD | Met criteria for ASD | Met criteria for ASD | Met criteria for ASD |
|  | SCQ | Above cut-off for ASD | Above cut-off for ASD | Above cut-off for ASD | Above cut-off for ASD | Above cut-off for ASD | Above cut-off for ASD |
|  | SRS-2 | Severe range | Severe range | Moderate range | Severe range | Severe range | Severe range |
| Anxiety symptoms^5^ | |  | Generalized anxiety diagnosis |  |  |  |  |
|  | RCADS | WNL^6^ | Clinical range | WNL^6^ | N/A^7^ | N/A^7^ | N/A^7^ |
|  | CBCL/ ABCL anxiety subscale | Borderline range | Clinical range | Clinical range | WNL^6^ | WNL^6^ | Clinical range |
| Attention concerns^8^ | | ADHD diagnosis | ADHD diagnosis |  |  |  |  |
|  | SWAN | Above cut-off for ADHD | Below cut-off for ADHD | Above cut-off for ADHD | Below cut-off for ADHD | N/A^7^ | N/A^7^ |
|  | CBCL/ABCL attention subscale | Clinical range | WNL^6^ | Borderline range | WNL^6^ | Clinical range | Clinical range |
| Emotional regulation concerns^9^ | |  |  |  |  |  |  |
|  | BRIEF | Clinical range | Clinical range | WNL^6^ | N/A | Clinical range | Clinical range |
|  | ABCL antisocial personality problems subscale | N/A | N/A | N/A | WNL^6^ | N/A | N/A |
| Externalizing behaviours^10^ | |  |  |  |  |  |  |
|  | CBCL/ ABCL Externalizing subscale | Clinical range | Borderline range | Borderline range | WNL^6^ | Clinical range | Clinical range |
| Obsessive compulsive tendencies | |  |  |  |  |  |  |
|  | TOCS^11^ | WNL^6^ | WNL^6^ | WNL^6^ | WNL^6^ | N/A^7^ | N/A^7^ |
| Depressive symptoms | |  |  |  |  |  |  |
|  | CDI 2/ BDI-II^12^ | WNL^8^ | WNL^6^ | WNL^6^ | WNL^6^ | N/A^7^ | N/A^7^ |

^1^IQ = Intelligence quotient, WASI-II = Wechsler Abbreviated Scale of Intelligence, Second Edition, WPPSI-IV = Wechsler Preschool and Primary Scale of Intelligence, Fourth Edition, SB-5 = Stanford-Binet Intelligence Scale, Fifth Edition

^2^ABAS-II = Adaptive Behaviour Assessment System, Second Edition

^3^OWLS-II = Oral and Written Language Scales, Second Edition, PPVT-4 = Peabody Picture Vocabulary Test, Fourth Edition, EVT-2 = Expressive Vocabulary Test, Second Edition

^4^ADOS-2 = Autism Diagnostic Observation Schedule, Second Edition, ADI-R = Autism Diagnostic Interview- Revised, SCQ = Social Communication Questionnaire, SRS-2 = Social Responsiveness Scale, Second Edition, ASD = Autism Spectrum Disorder

^5^RCADS = Revised Children’s Anxiety and Depression Scale, CBCL = Child Behaviour Checklist, ABCL = Adult Behaviour Checklist

^6^WNL = within normal limits

^7^Information not available because proband was too young to be assessed

^8^SWAN = Strengths and Weaknesses of ADHD Symptoms and Normal Behaviour Rating Scales, ADHD = Attention Deficit and Hyperactivity Disorder, CBCL = Child Behaviour Checklist, ABCL = Adult Behaviour Checklist

^9^BRIEF = Behaviour Rating Inventory of Executive Function, ABCL = Adult Behaviour Checklist

^10^CBCL = Child Behaviour Checklist, ABCL = Adult Behaviour Checklist

^11^TOCS = Toronto Obsessive Compulsive Scale

^12^CDI 2 = Children’s Depression Inventory 2, BDI-II = Beck Depression Inventory

**Supplementary Table 3: *De novo* missense variants in the CXXC zinc finger domain of *KMT2A* individuals with WSS and NDDs**

| p.HGVS | Number of patients ascertained for WSS | Number of patients ascertained for NDD |
| --- | --- | --- |
| p.Arg1154Trp | 1 | 2 |
| p.Cys1155Tyr | 2 | 1 |
| p.Cys1161Gly | 1 | 0 |
| p.Gly1168Asp | 2 | 0 |
| p.Gly1181Asp | 1 | 0 |
| p.Lys1186Glu | 0 | 1 |
| p.Cys1189Tyr | 1 | 0 |
| p.Cys1194Tyr | 0 | 1 |


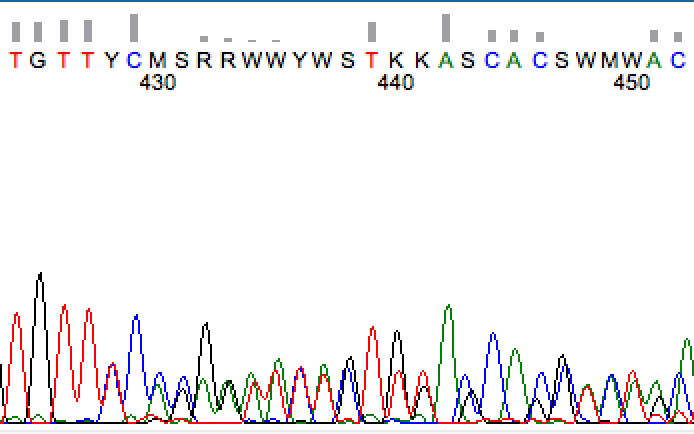


Patient 3


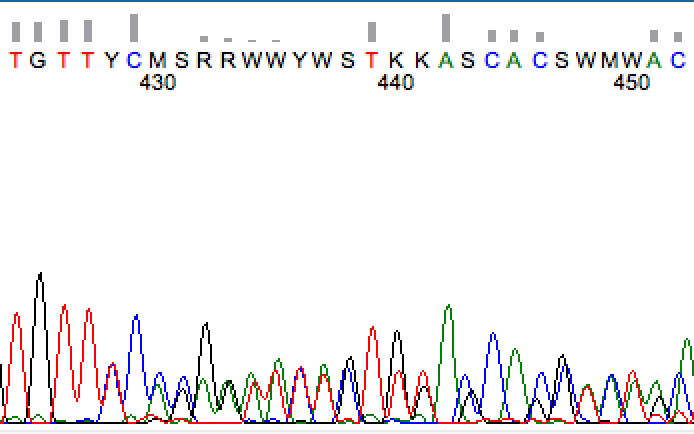

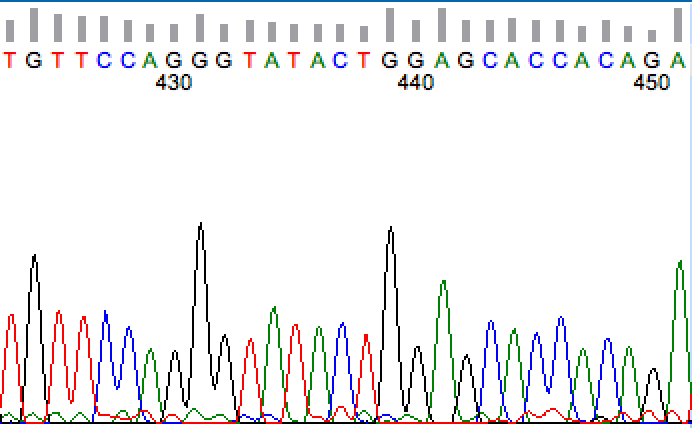


Father


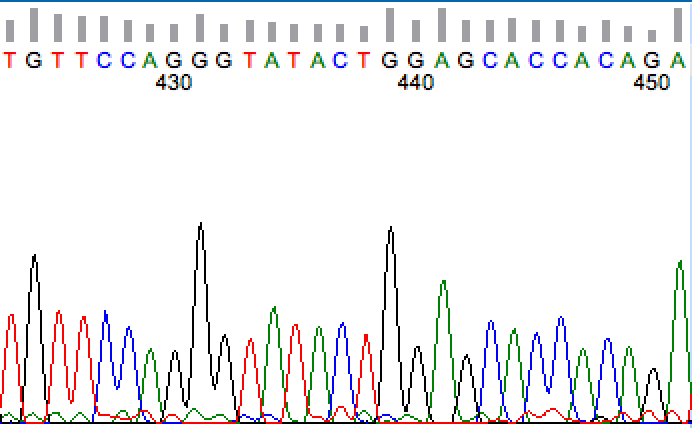

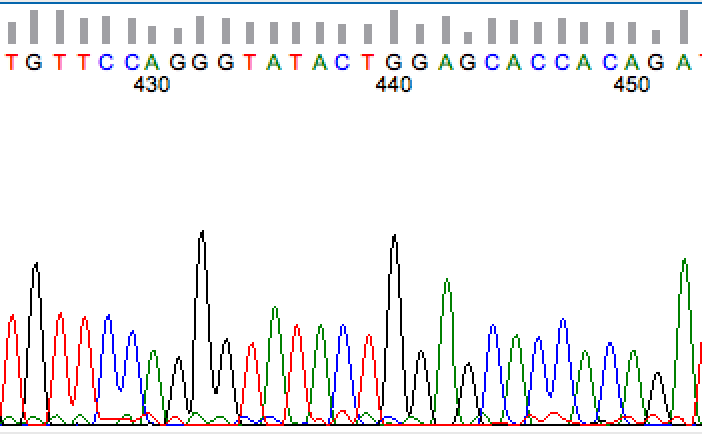


Mother


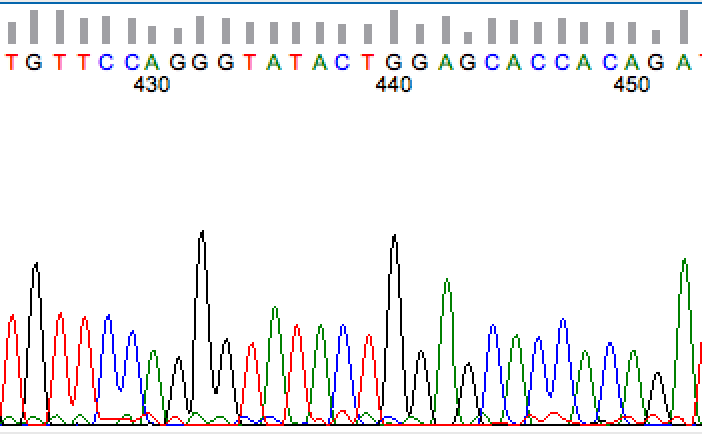

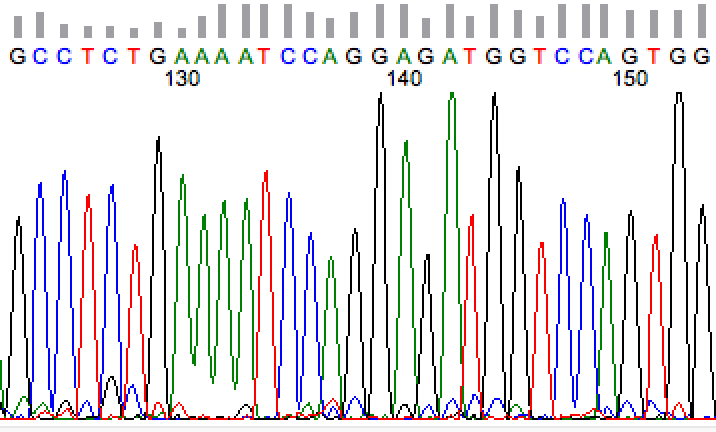


Father


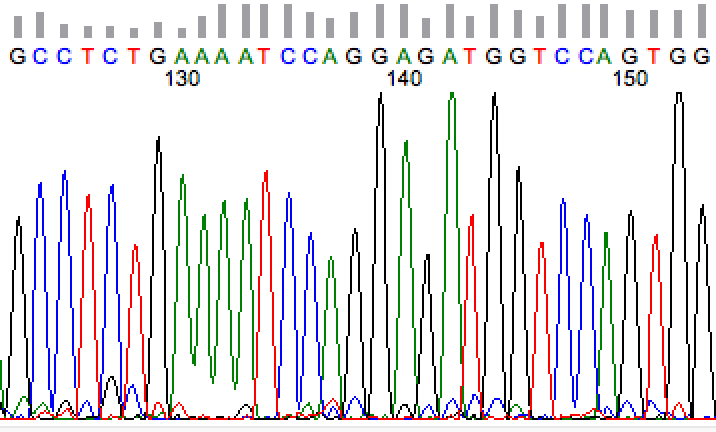

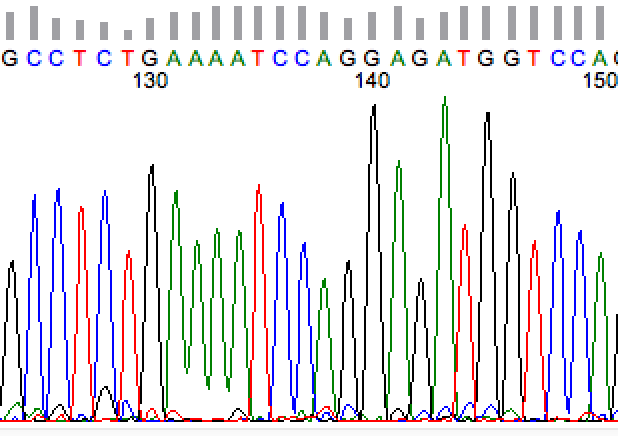

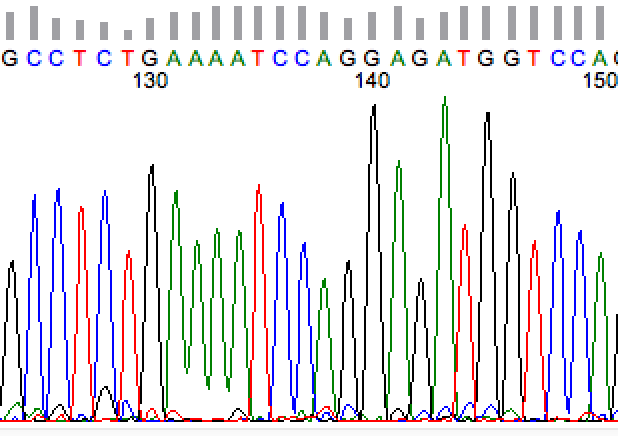


Mother


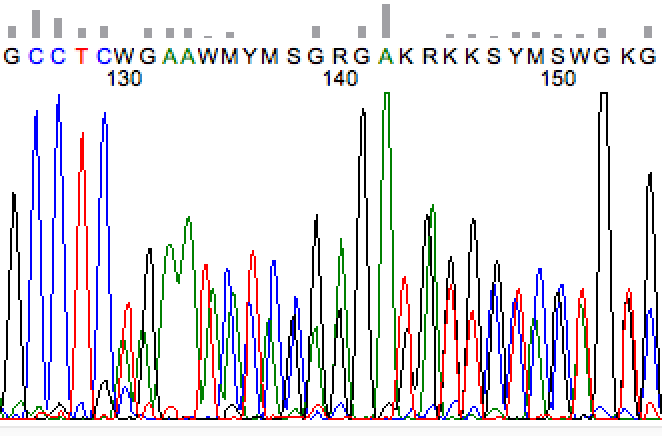

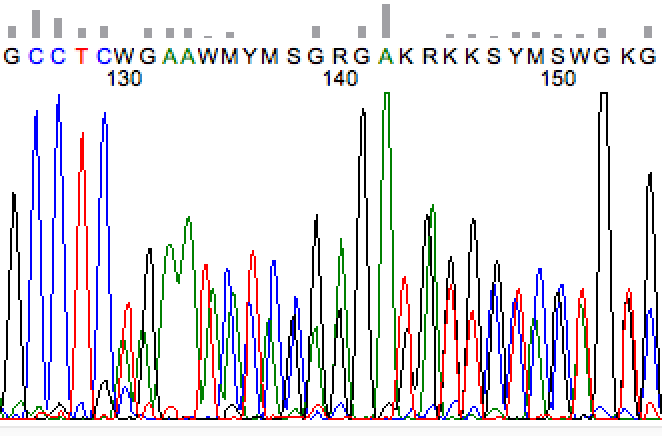


Patient 4


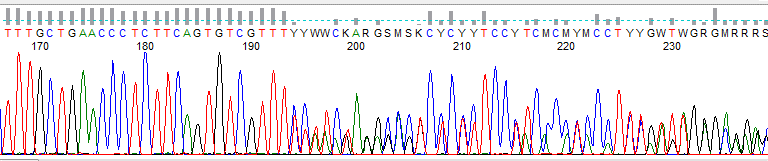

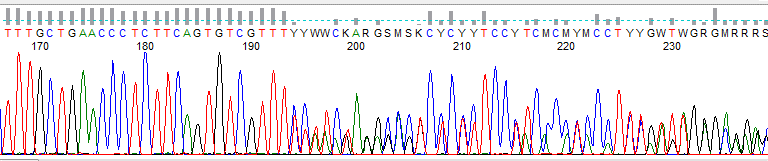


Patient 2


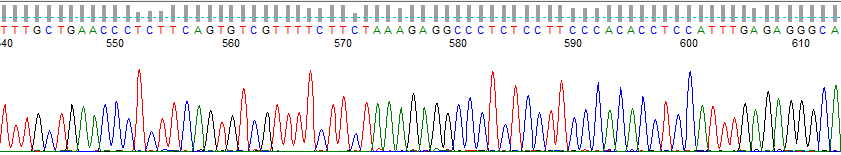

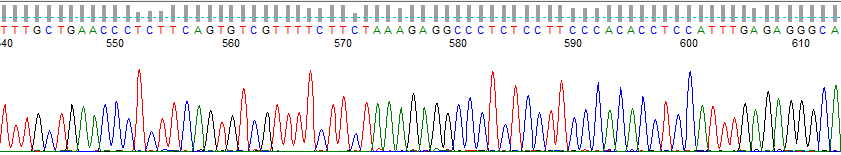


Mother


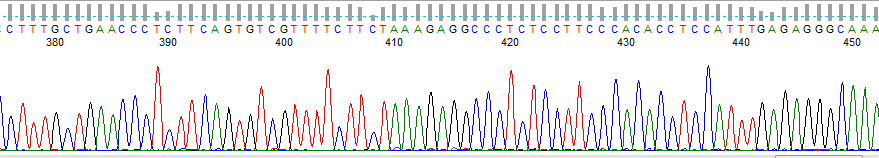

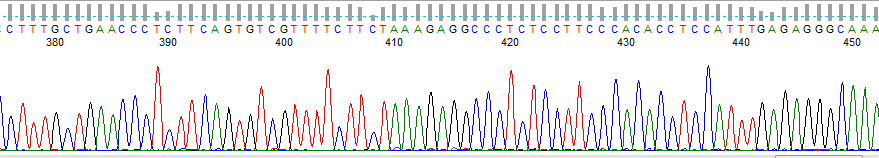


Father


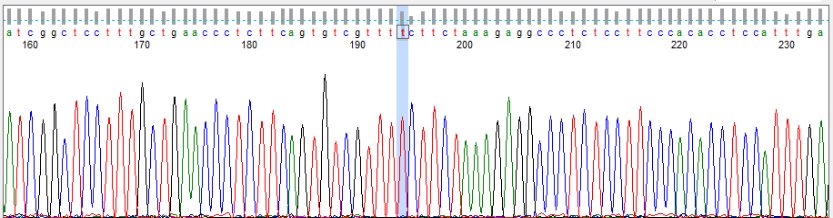

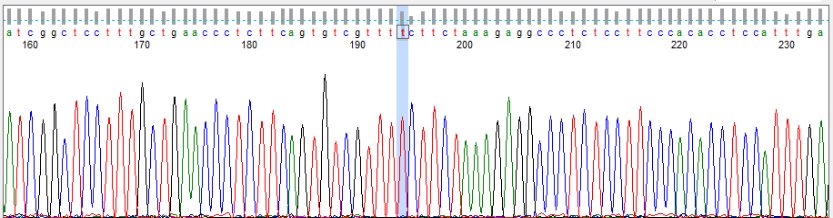


Sibling

Patient 3

Patient 4

Patient 2


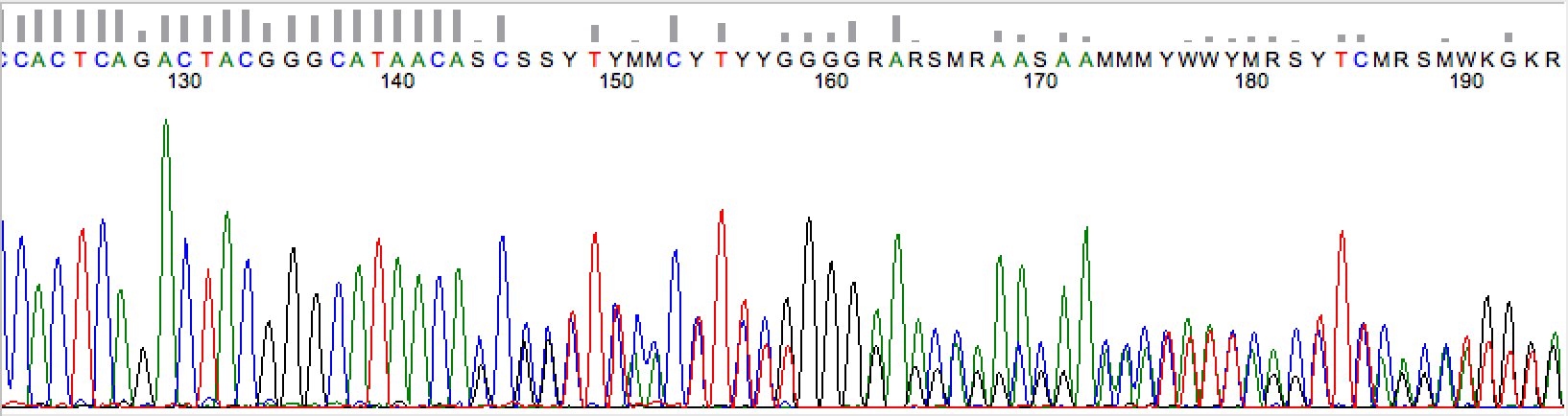

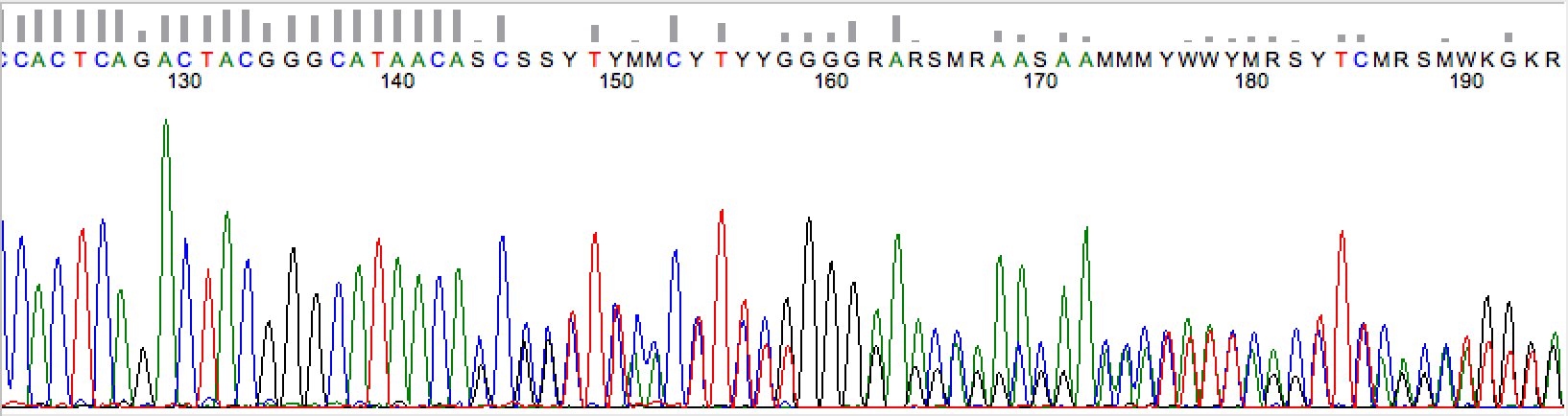

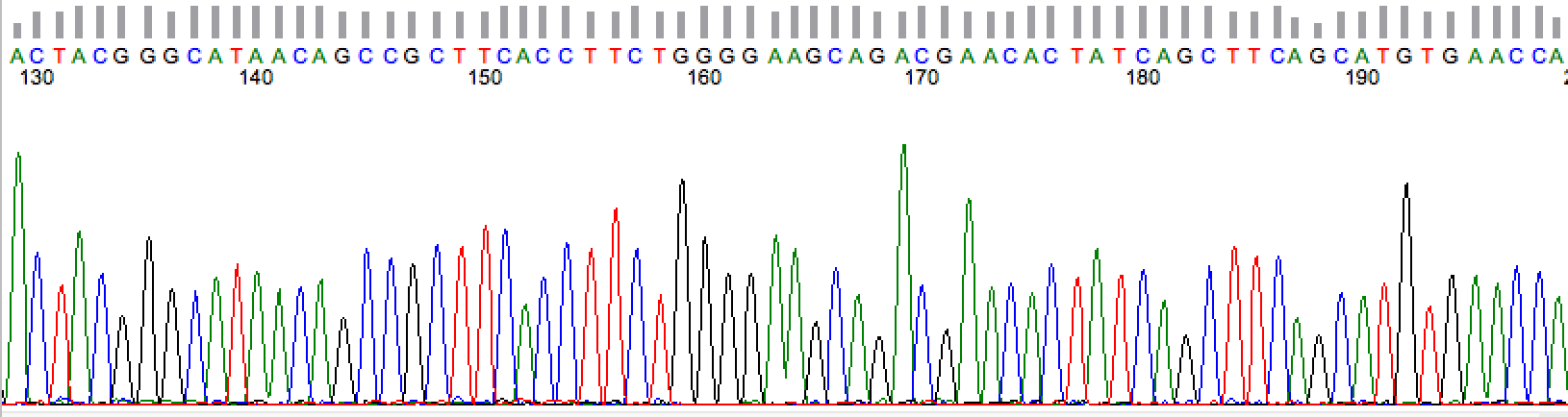

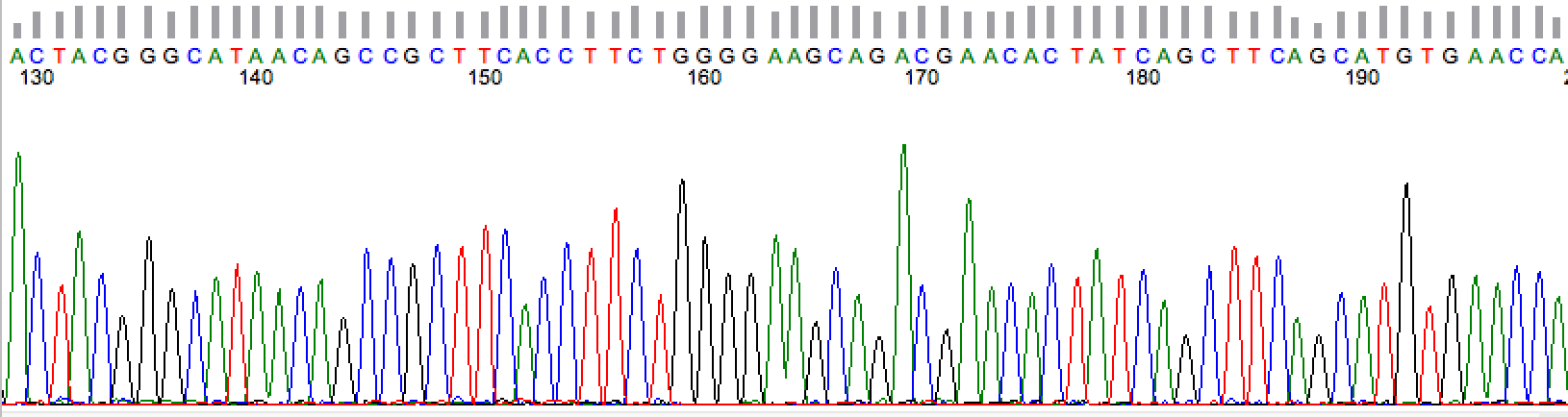

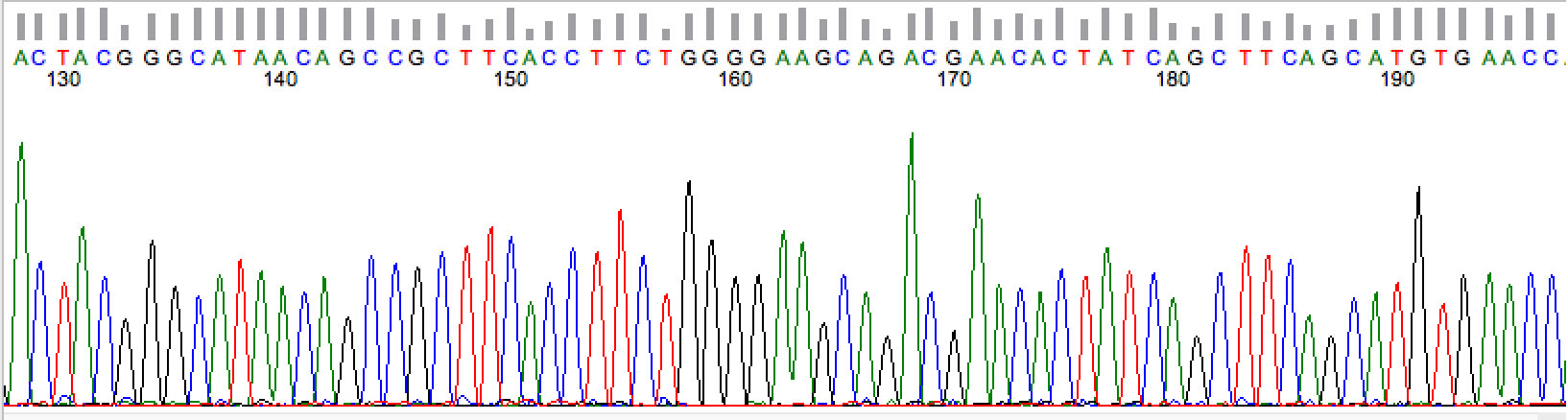

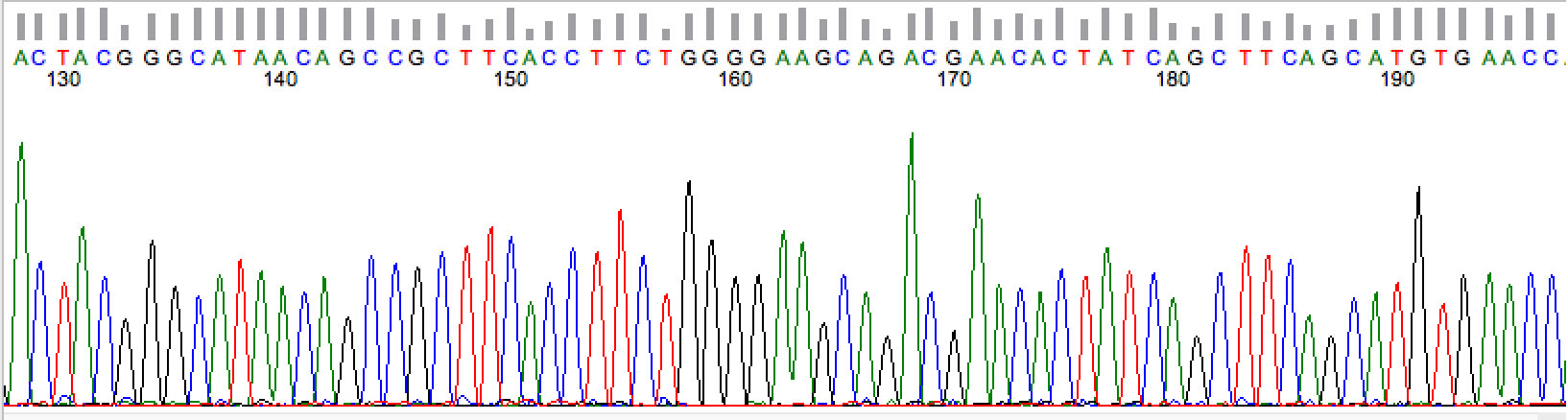


Patient 1

Father

Mother

Patient 1

**Supplementary Figure 1: Sanger sequencing validation of *de novo* mutations in *KMT2A* for patients 1-4.** Patient 6 had targeted sequencing for *KMT2A.* Sanger sequencing validation data for patients 5 and 6 are not available because they underwent clinical diagnostic testing (WES trio analysis and targeted sequencing, respectively).


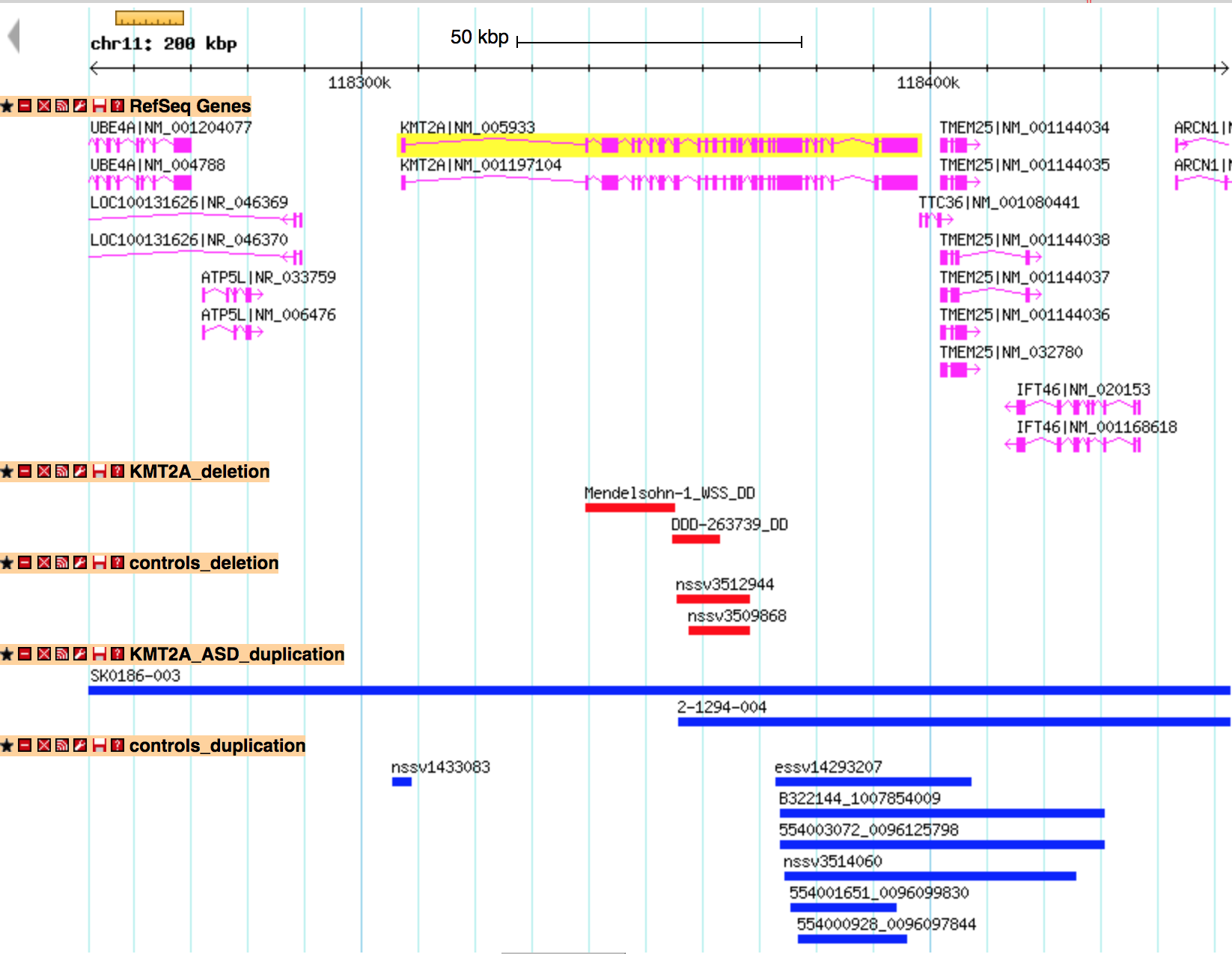


**Supplementary Figure 2: CNVs overlapping *KMT2A* in individuals with ASD, WSS, and/or developmental delay and controls.** Deletions and duplications are shown in red and blue respectively. The second and third panels shows the deletions found in individuals with WSS^1^ or developmental delay^2^ and controls^3,4^, respectively. The fourth and fifth panels shows the gains found in individuals with ASD and controls, respectively.

**Supplementary References**

1 Mendelsohn, B. A., Pronold, M., Long, R., Smaoui, N. & Slavotinek, A. M. Advanced bone age in a girl with Wiedemann-Steiner syndrome and an exonic deletion in KMT2A (MLL). *Am J Med Genet A* **164A**, 2079-2083, doi:10.1002/ajmg.a.36590 (2014).

2 Deciphering Developmental Disorders, S. Prevalence and architecture of de novo mutations in developmental disorders. *Nature* **542**, 433-438, doi:10.1038/nature21062 (2017).

3 MacDonald, J. R., Ziman, R., Yuen, R. K., Feuk, L. & Scherer, S. W. The Database of Genomic Variants: a curated collection of structural variation in the human genome. *Nucleic Acids Res* **42**, D986-992, doi:10.1093/nar/gkt958 (2014).

4 Zarrei, M., MacDonald, J. R., Merico, D. & Scherer, S. W. A copy number variation map of the human genome. *Nat Rev Genet* **16**, 172-183, doi:10.1038/nrg3871 (2015).
